# The Spectrum of Fundus Autofluorescence Findings in Birdshot Chorioretinopathy

**DOI:** 10.1155/2009/567693

**Published:** 2010-02-08

**Authors:** GianPaolo Giuliari, David M. Hinkle, C. Stephen Foster

**Affiliations:** ^1^Massachusetts Eye Research and Surgery Institution, Cambridge, MA 02142, USA; ^2^Ocular Immunology and Uveitis Foundation, 5 Cambridge Center, Cambridge, MA 02142, USA; ^3^Harvard Medical School, Boston, MA 02115, USA

## Abstract

*Objective*. To describe the diverse patterns observed with the use of autofluorescence fundus photography (FAF) in patients with Birdshot chorioretinopathy (BSCR). *Methods*. A chart review of patients with BSCR seen at the Massachusetts Eye Research and Surgery Institution, who had autofluorescence fundus photography. The data obtained included age, gender, presence of the HLA-A29 haplotype, and current treatment. *Results*. Eighteen eyes with HLA-A29 associated BSCR were included. Four eyes presented with active inflammation. Correspondence of the lesions noted in the colour fundus photograph was observed in 3 eyes which were more easily identified with the FAF. Fifteen eyes had fundus lesions more numerous and evident in the FAF than in the colour fundus photograph. *Conclusion*. Because FAF testing provides valuable insight into the metabolic state of the PR/RPE-complex, it may serve as a useful noninvasive assessment tool in patients with posterior uveitis in which the outer retina-RPE-choriocapillaries-complex is involved.

## 1. Introduction

Birdshot chorioretinopathy (BSCR) is an uncommon cause of chronic posterior uveitis, most frequently affecting Caucasian women in the fifth and sixth decades of life [1, 2]. The most common clinical signs are vitritis and multiple bilateral, hypopigmented inflammatory lesions in the post-equatorial fundus [3–5].

The aetiology of BSCR remains unclear, although a relationship exists with the human leukocyte antigen (HLA) -A29; therefore, an autoimmune mechanism has been proposed [5–7].

Due to the variety of clinical manifestations observed in BSCR, the diagnosis may be elusive, especially early in the course of the disease, prior to the evolution of the characteristic hypopigmented spots in the fundus [8, 9]. Additionally, the diffuse pigmentary loss noted through time in the typical choroidoretinal lesions of BSCR is not a reliable marker of disease activity. Furthermore, some studies suggest that the retinal vascular leakage observed by fluorescein angiography (FA) may not be sufficient to decide on treatment [10].

Different techniques and ancillary testing are crucial in patients with BSCR, not only for diagnostic purposes but to guide adequate follow up and treatment monitoring as well. An extensive clinical history and ophthalmic examination accompanied by fundus photography along with FA and electroretinography, remain invaluable to the management of these patients.

Fundus autofluorescence photography (FAF) is a relatively new retinal assessment technique which capitalizes on the presence of lipofuscin, an important substance found in the retinal pigment epithelial (RPE) cells, allowing us to obtain unique information about the structure and function of the RPE [11]. FAF may better assess the presence of the typical inflammatory lesion observed in fundus of patients with BSCR, and facilitate its visualization in patients with blond fundus.

## 2. Methods

This retrospective case series study was approved by the Institutional Review Board of the Massachusetts Eye and Ear Infirmary, and adheres to the tenets of the Declaration of Helsinki. All patients with BSCR seen at the Massachusetts Eye Research and Surgery Institution (MERSI), from July 2008 to October 2008, who underwent FAF imaging, were included in the analysis. Those patients in whom macular oedema was clinically suspected, the central macular thickness (CMT) was recorded by time domain optical coherence tomography (OCT-Stratus III, Carl Zeiss, Meditec, Dublin, CA).

Data were obtained through review of the electronic medical records of each patient (NextGen, Horsham, PA) and entered into Excel (Microsoft Corporation, Redmond, Washington) data entry worksheets. General data included age, gender, presence of the HLA-A29 haplotype, and current therapeutic regimen. To evaluate the clinical status of the disease, a complete ophthalmic examination was performed with the use of slit lamp biomicroscopy, noncontact fundus lens, and indirect ophthalmoscopy. The ocular inflammatory status was judged according to the Standardization of Uveitis Nomenclature (SUN) Working Group criteria [12], as well as findings on the FA. Active inflammation was defined as ≥1+ anterior chamber cell/flare and/or ≥1+ vitreous haze. FA signs considered indications of active disease included hyperfluorescence of the optic disc, leakage of dye from capillaries and venules during the late phase, and delayed arterial filling time.

FAF was performed using a fundus camera (Topcon Medical Systems, Paramus, NJ—Spectrotech) with an excitation filter peak at 560 nm (bandwidth, 535–585 nm) and a barrier filter centered at 655 nm (bandwidth, 605–705 nm).

## 3. Results

Eighteen eyes of 9 patients met the inclusion criteria and were included in the analysis. All patients had the HLA-A29 haplotype. The mean age was 55.5 years (range 48–69 years). Two patients (22%) were males. The median visual acuity was 20/25 (range 20/20–20/50).

The most common treatment regimen employed was mycophenolate mofetil, and cyclosporine alone or combined in five (56%) patients. Daclizumab was employed in two patients; adalimumab and intravenous immunoglobulin were employed in one patient each. One patient was taking oral corticosteroids (Table 1).

**Figure 1:**
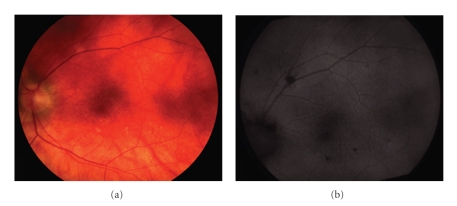
Images from the left eye of a 58-year-old woman with birdshot chorioretinopathy (patient 3). (a) Fundus colour photograph shows poorly pigmented fundus with scatter BSCR around the optic nerve, superior arcade, and inferior to the fovea. (b) Autofluorescence photograph shows scattered areas of RPE atrophy as hypoautofluorescent spots correspondent to the BSCR noted in the colour photograph.

**Figure 2:**
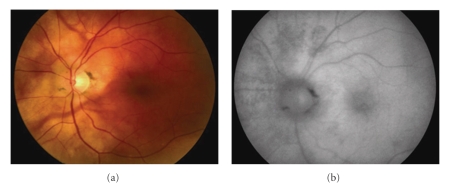
Images from the left eye of a 58-year-old woman with birdshot chorioretinopathy (patient 5). (a) Fundus colour photograph shows poorly pigmented fundus with scatter BSCR lesions nasal to the optic nerve. (b) Autofluorescence photograph shows scattered areas of RPE atrophy as hypoautofluorescent spots and as a linear pattern along the retinal vessels.

Four (22%) eyes presented with active inflammation characterized by optic nerve leaking and/or retinal vasculitis assessed by FA. Three (17%) eyes presented with macular oedema confirmed both by FA and OCT. None of the eyes with macular oedema showed macular hypoautofluorescence. Full correspondence of the lesions noted in the colour fundus photograph was observed in 3 (17%) eyes of 2 patients; however, they were more easily identified with the FAF. In 15 eyes (83%), fundus lesions were more numerous and more easily identified in the FAF than in the colour fundus photograph. These fundi had the typical BSCR lesions, peripapillary chorioretinal atrophy characterized as a circumferential hypoautofluorescence around the optic nerve, and in 3 (17%) eyes a linear hypoautofluorescence pattern along the retinal vessels was noted (Figures 1 and 2).

## 4. Discussion

Despite recent advances in diagnostic imaging, the diagnosis of BSCR remains a challenge. OCT is extremely valuable in the quantification and monitoring of macular oedema, the most common complication resulting in vision loss in BSCR [5, 6, 10, 11, 13]. Despite the limitations of FA, it remains an important tool to assess the status of the retinal vasculature [10]. These techniques are augmented by full field electroretinogram (ERG) in which an important correlation between the progression of the disease has been found with the flicker implicit time and bright scotopic B-wave amplitude [14–16]. Indocyanine green angiography and visual field testing (especially with SITA-SWAP protocol) are valuable tools in the care and longitudinal monitoring of these patients with BSCR [17, 18].

More recently, FAF, a relatively new technique, has been employed to evaluate the characteristics of the chorioretinal lesions in BSCR [19]. This new technology was first used in the care of patients with dry age related macular degeneration, and more recently in patients with different types of posterior uveitis including multifocal choroiditis and BSCR [20, 21].

**Figure 3:**
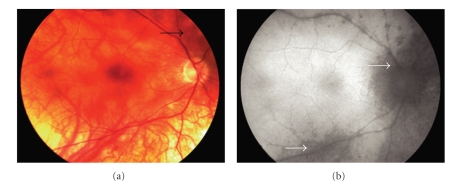
Images from the right eye of a 69-year-old woman with birdshot chorioretinopathy (patient 6). (a) Fundus photograph shows a poorly pigmented fundus with scattered lesions nasally and superior of the optic nerve (black arrow). (b) Autofluorescence photograph demonstrates circumferential hypoautofluorescence around the optic nerve, scattered multiple multifocal hypoautofluorescent spots, and a linear hypoautofluorescence pattern along the retinal vessels (white arrows).

We noted a wide spectrum of FAF patterns in our cohort. The typical BSCR fundi lesions did not necessarily correspond to the areas of RPE atrophy shown with the FAF. In 3 eyes, full correspondence of the BSCR lesions was observed. The remaining eyes (15 eyes) showed more areas of RPE atrophy in the FAF than in the colour photograph. Lesions were significantly easier to identify in the FAF than in the colour photograph, especially in those eyes with light fundus pigmentation in which the visualization of BSCR lesions can be challenging. Figure 3 shows the right eye of a 69-year-old female with a well-confirmed BSCR diagnosis of almost 2 years, with HLA-A29 haplotype. At the moment of the FAF, she presented with a visual acuity of 20/25 and was in treatment with intravenous immunoglobulin. Note in the colour photograph (Figure 3(a)) the hypopigmented lesions nasal to the optic nerve and around the arcades; however due to the poor fundus pigmentation, these lesions are difficult to visualize. Note the striking difference with the FAF in which areas of RPE atrophy are significantly more numerous and clear than in the colour photograph. A perivascular linear FAF pattern shown in Figures 2 and 3 was also noted. This finding was previously described by Koizumi et al. and led his group to postulate a possible role of inflammation induced damage of the RPE [19].

The origin of BSCR lesions is controversial. Some authors support the idea that birdshot is a chorioretinopathy, while others believe that it is a retinochoroidopathy [22]. This lack of correspondence between the colour fundus photograph and the FAF, as suggested by other authors, may indeed be explained by two separate mechanisms of damage of the choroid and the RPE, which may vary in each patient [19].

FAF testing provides valuable insight into the metabolic state of the PR/RPE complex; we find it useful as a noninvasive diagnostic tool in patients with posterior uveitis, when the outer retina-RPE choriocapillaries complex is involved. Limitations for this study include the small sample size and the inherent bias of its retrospective nature.

## Figures and Tables

**Table 1:** Demographic characteristics.

Patients	Eye	Age	Gender	Diagnosis*	HLA A29	Treatment	VA	ERG**	OCT***
1	OD	50	Female	2001	Positive	Daclizumab	20/20	38	241
	OS						20/30	30	383

2	OD	48	Male	1997	Positive	MMF+CSA	20/50	35	562
	OS						20/20	30.5	241

3	OD	58	Female	2008	Positive	MMF+CSA	20/30	34.5	
	OS						20/25	31.5	

4	OD	59	Male	1998	Positive	MMF+CSA	20/20	35.7	
	OS						20/25	31.9	

5	OD	58	Female	2004	Positive	MMF+CSA	20/25	36.8	165
	OS						20/40	44.5	312

6	OD	69	Female	2007	Positive	IVIG	20/25	32.7	
	OS						20/30	31.8	

7	OD	53	Female	2006	Positive	MMF+CSA	20/20	29	
	OS						20/20	27.8	

8	OD	56	Female	2005	Positive	Daclizumab	20/20	31.5	213
	OS						20/20	30.5	217

9	OD	49	Female	2005	Positive	Adalimumab	20/20	27.5	
	OS					Prednisone	20/20	26	

*Year in which the diagnosis was made; **Implicit times (ms); ***Central macular thickness (microns).

VA: Best corrected visual acuity; ERG: electroretinogram; OCT: optical coherence tomography; MMF: mycophenolate mofetil; CSA: cyclosporine; IvIg: intravenous immunoglobulin.
